# Myrosinase-dependent and –independent formation and control of isothiocyanate products of glucosinolate hydrolysis

**DOI:** 10.3389/fpls.2015.00831

**Published:** 2015-10-06

**Authors:** Donato Angelino, Edward B. Dosz, Jianghao Sun, Jennifer L. Hoeflinger, Maxwell L. Van Tassell, Pei Chen, James M. Harnly, Michael J. Miller, Elizabeth H. Jeffery

**Affiliations:** ^1^Department of Food Science and Human Nutrition, University of Illinois at Urbana-Champaign, Urbana, IL, USA; ^2^Food Composition and Methods Development Laboratory, Beltsville Human Nutrition Research Center, Agricultural Research Service, U.S. Department of Agriculture, Beltsville, MD, USA

**Keywords:** isothiocyanate, myrosinase, glucosinolate hydrolysis, microbiome, thiol binding, sulforaphane

## Abstract

Brassicales contain a myrosinase enzyme that hydrolyzes glucosinolates to form toxic isothiocyanates (ITC), as a defense against bacteria, fungi, insects and herbivores including man. Low levels of ITC trigger a host defense system in mammals that protects them against chronic diseases. Because humans typically cook their brassica vegetables, destroying myrosinase, there is a great interest in determining how human microbiota can hydrolyze glucosinolates and release them, to provide the health benefits of ITC. ITC are highly reactive electrophiles, binding reversibly to thiols, but accumulating and causing damage when free thiols are not available. We found that addition of excess thiols released protein-thiol-bound ITC, but that the microbiome supports only poor hydrolysis unless exposed to dietary glucosinolates for a period of days. These findings explain why 3–5 servings a week of brassica vegetables may provide health effects, even if they are cooked.

## Introduction

Isothiocyanates (ITC) are highly reactive products of the myrosinase-glucosinolate defense system found throughout the order Brassicales (Verkerk et al., 2009). ITC deter or even kill both generalist insects feeding on *Brassicaceae* and a number of pathogens that attack the plant, acting as natural pesticides (Kissen et al., 2009). Yet the parent glucosinolates exert little or no activity until they are hydrolyzed, suggesting an absolute requirement for hydrolysis for this host defense (Angelino and Jeffery, 2014). ITC even elicit a strong host-defense response in people, resulting in prevention of chronic disease, including cancer and cardiovascular disease. Upon eating a raw brassica meal, ITC metabolites appear in the blood (Conaway et al., 2000). But when brassica are heat-treated, almost no ITC appear in blood (Conaway et al., 2000). For example, in one study plasma sulforaphane and metabolites in those eating cooked broccoli were one tenth that of those eating raw broccoli (Vermeulen et al., 2008); in another study, there was less than 1% sulforaphane and metabolites compared to those eating only slightly cooked broccoli sprouts still containing active myrosinase (Saha et al., 2012). These data suggest that plant myrosinase is essential for an effective dose of ITC to be formed and absorbed, supporting the traditional idea that cooked brassica are less likely than uncooked brassica to provide health benefits (Steinmetz and Potter, 1991). Yet *ex vivo* studies show that glucosinolate hydrolysis by the microbiome is possible, although activity appears to be far less than by myrosinase and is possibly limited to a few specific commensal bacteria, *ex vivo* (Luang-In et al., 2015). The choice of preparation techniques for broccoli feeding studies may impact estimation of sulforaphane bioavailability. In different raw broccoli preparations where glucoraphanin levels were similar, there was 58% bioavailability from freeze-dried, rehydrated ground raw broccoli, but only 33% from freeze-dried, rehydrated whole florets (Oliviero et al., 2014). When the whole florets were cooked to destroy myrosinase prior to freeze-drying and rehydrating, bioavailability dropped, but only to about one third that of raw broccoli floret bioavailability. These data suggest that floret structure may impact bioavailability. However, none of the kinetic studies show sufficient absorption from cooked broccoli to explain why epidemiological data show health benefits from individuals eating cooked brassica several times a week. Studies are needed to determine how cooked broccoli can be effective. The aim of this perspective is to raise questions about enhancing glucosinolate hydrolysis by the microbiome and what happens to reactive ITC formed by gut bacteria. We will discuss how the choice of analytical methods used, so that both tissue-bound ITC and free ITC are estimated, may provide further insight into myrosinase-independent glucosinolate hydrolysis.

## Glucosinolate Hydrolysis

The glucosinolate substrates of myrosinase are a broad family of S-glucosides that, upon hydrolytic cleavage of the sulfur-glucose bond, release glucose and form an unstable aglycone intermediate that rearranges to form one of a number of different products: a highly bioactive ITC, a nitrile, thiocyanate or cyclic compounds (Halkier and Gershenzon, 2006). This depends on the specific glucosinolate, in some cases the specific myrosinase, and often the presence or absence of myrosinase binding proteins and myrosinase-associated proteins (Kuchernig et al., 2012). In the absence of any myrosinase-associated proteins, only ITC can be formed. The simple nitriles, promoted by low pH and iron or by the myrosinase binding protein epithiospecifier protein (ESP), appear to be the least bioactive. However, adverse effects are dose-dependent for all products and nitriles, particularly epithionitriles, are associated with toxicities in livestock (Wallig et al., 1988; Collett et al., 2014). Yet molecule for molecule, the greatest reactivity/toxicity is observed with ITC. For this reason, plants, insects, bacteria and humans have evolved multiple ways to bind ITC or redirect hydrolysis away from ITC formation in an attempt to avoid toxicity (Wittstock et al., 2003).

## Myrosinase Isoenzymes

Myrosinase (EC 3.2.1.147) specifically cleaves thio-linked glycosides. Myrosinases from *Arabidopsis thaliana* (subfamilies AtTGG1–AtTGG6) and *Brassica napus* (MA, MB, and MC) are best characterized (Nong et al., 2010). The latter are also found in *Brassica oleracea*, L. var. italica, the popular vegetable broccoli. These two nomenclatures are related phylogenetically, as part of the glycoside hydrolase superfamily, found across tissues and species of the order Brassicales, as MYR I (including MA, MB, MC, AtTGG1-3) and MYR II (including AtTGG4 and 5 and others). Most MYR I are present in above-ground tissues, whereas MYR II enzymes are most often in root tissue (Andersson et al., 2009; Kissen et al., 2009). Distinct patterns of expression suggest that the different myrosinase enzymes may have different roles, such as differences in substrate specificity, since there is a different but overlapping glucosinolate expression between root and above-ground tissue in many species (Rosa and Rodrigues, 1998; Kastell et al., 2013). Recently, a different protein with myrosinase activity, PEN2, was found to play a role where, in living tissue, it travels to stomata, where it hydrolyzes indole glucosinolates, the products of which then attack fungal infestation at the leaf entrance (Bednarek et al., 2009).

## Protection From Isothiocyanates by Different Species

The plant protects against inadvertently triggering the glucosinolate-myrosinase system. Myrosinases are compartmentalized in myrosin cells, away from their glucosinolate substrates, so that hydrolysis cannot occur. When tissue is damaged, myrosinase and glucosinolates are brought together (Kissen et al., 2009). Also, myrosinase-associated proteins may direct hydrolysis either toward or away from ITC formation.

Specialist insects have developed a number of unique mechanisms to defuse the “bomb” (Winde and Wittstock, 2011). The cabbage white butterfly larva (*Pieris* sp.) has evolved a nitrile-specifier protein to direct hydrolysis away from ITC formation, toward the relatively non-toxic nitrile. Interestingly, this protein bears no relationship to the ESP present in brassica that diffuses the reaction in a similar manner. Phloem-sucking insects avoid myrosin cells and thus avoid glucosinolate hydrolysis. The aphid *Brevicoryne brassicae* is one of the most intriguing examples, since it not only stores intact glucosinolates, but synthesizes and stores separately its own myrosinase-like enzyme. Thus, when predators chew into *Brevicoryne brassicae*, the glucosinolates are hydrolyzed, leading to this aphid being named “walking mustard oil bomb” (Winde and Wittstock, 2011).

A number of insects contain a functional glutathione (GSH)-*S*-transferase enzyme, similar in action to that found in mammals, which conjugates ITC to GSH, rendering ITC temporarily harmless. Unlike eukaryotes and most Gram-negative bacteria, many Gram-positive bacteria have no GSH—but synthesize alternative low molecular weight thiols, such as bacillithiol, which also function together with a thiol transferase enzyme to bind reactive xenobiotic electrophiles like ITC (Fang et al., 2013).

In mammals, ITC toxicity is only seen when, as with specialist insects, brassica constitute the major part of their diet. Feeding defatted brassica seed cake to livestock as a protein source, has been a consistent problem with rape (*B. napus* L. spp. *oleifera*), a rich source of progoitrin, causing reproductive and thyroid problems unless the rapeseed is bred to be low in glucosinolates (Jackson, 1969). In humans, very few adverse effects from ingesting brassica have been recorded, possibly because humans eat a varied diet, so that glucosinolate hydrolysis products typically do not reach toxic levels. At these low levels, ITC trigger a health-defense system in mammals. The key trigger is considered accumulation and transport to the nucleus of an otherwise cysteine-bound transcription factor, nuclear factor (erythroid-derived 2)-like 2, or Nrf2. This transcription factor upregulates a broad range of xenobiotic metabolizing enzymes, anti-oxidant enzymes and others, including GSH-*S*-transferases.

## Quenching the Reactivity of Isothiocyanates

Isothiocyanates are highly reactive electrophiles, which damage living systems by binding and disrupting tissues. First, or at low concentration, they bind reversibly to thiols: free thiols like GSH as well as free sulfhydryls on cysteines in proteins. This could lead to specific protein sites of ITC attack, for example binding to the cysteine in the ATP binding site of kinases, proteins that have key regulatory roles in cellular metabolism across species (Cross et al., 2007). However, because ITC binding is reversible, an excess of GSH or other free thiol could release the protein-bound ITC. It is interesting to note here that in mammals ITC trigger the synthesis of GSH, enhancing the intracellular free GSH concentration and leading to greater expulsion of thiol-bound ITC. Studies with mammalian cell culture show that once ITC are bound to GSH, the conjugate undergoes efflux from the cell via the multidrug resistance transporters. In this static system of cell culture, the conjugate breaks down in the medium and releases free ITC that re-enters the cell (Callaway et al., 2004). This not only depletes the cell of GSH, but when all the GSH has been depleted, the ITC binds to cellular proteins and accumulates to very high concentrations. Whether this happens in the dynamic system of the whole body, where the effluxed conjugate will leave the site in the bloodstream, has yet to be determined. However, the few studies reporting tissue levels of sulforaphane do not report levels much higher than those reported for plasma (Cornblatt et al., 2007). When cells undergo oxidative stress, some proteins become glutathiolated at their free cysteines. This could potentially alter the pattern of any subsequent ITC binding.

With about a 1000-fold less affinity than for thiols, ITC will bind to other cellular nucleophiles such as prolines and lysines within proteins (Hanschen et al., 2012). In contrast to thiol-binding, this is irreversible. Protein synthesis is required to replace the damaged protein and the ITC cannot be recovered. Measurement systems in use today cannot estimate these irreversibly bound ITC.

## Microbial Hydrolysis of Glucosinolates

Brassica have been used successfully as natural pesticides, killing many soil-borne bacteria, fungi and other plant pathogens (Smolinska et al., 1997). Those studies evaluate the impact of exogenously formed ITC as anti-bacterial agents. Whether gut bacteria can provide bioactive ITC in the absence of myrosinase is a very different question: the hypothesis under debate is that glucosinolates are hydrolyzed by the gut microbiota, then released for transport across the gut wall and enter into the mammalian circulation to provide health benefits. Thus three major questions arise: (i) do bacteria have sufficient hydrolyzing activity? (ii) are the resulting ITC bioavailable? and (iii) what is the chemical form of the ITC that permits safe transport? The first is an active area of enquiry, the second shows too little ITC absorption to be sure of causing health benefits and the third is assumed to be as glutathione metabolites, but no work exists on possible rate limitations at this step, or function of bacteria that do not make glutathione.

To evaluate hydrolysis of glucosinolates in mammals in the absence of myrosinase, we purified glucoraphanin from broccoli seed and administered it to rats either orally (by gavage directly into the stomach) or by injection into the abdomen/peritoneal cavity, which bypasses the gut microbiome (Bheemreddy and Jeffery, 2007). Following the oral dose, urinary sulforaphane and metabolites accounted for ∼20% of the dose. Although we also found a small amount of sulforaphane present following intraperitoneal administration, this was less than 5% of the dose and was obliterated by tying off the bile duct, suggesting that any sulforaphane formed in the absence of myrosinase requires the gut microbiome. This confirms clinical studies, that although the microbiome can hydrolyze glucosinolates, only small, potentially sub-threshold levels of hydrolysis products are detectable following a single meal of cooked brassica vegetables (Conaway et al., 2000; Saha et al., 2012).

In studies of human fecal or rat caecal microbiota incubated with glucosinolates *ex vivo*, there has frequently been little or no ITC product reported (reviewed in Angelino and Jeffery, 2014). Most researchers evaluating microbial glucosinolate hydrolysis in cultured bacteria have reported disappearance of glucosinolates, but only a small amount of erucin nitrile and essentially no sulforaphane formed from glucoraphanin. One report makes a strong argument that nitriles, not ITC, may be the end products of glucosinolate hydrolysis by the microbiome, suggesting that nitrile bioactivity should be further evaluated (Mullaney et al., 2013). In contrast, one study utilized a dynamic system that allowed product to cross dialysis tubing after formation and found, for the first time, significant ITC formation (Krul et al., 2002). We also developed a “dynamic” system, by incubating glucosinolates *in situ* in the ligated rat caecum and collecting portal blood (Lai et al., 2010). However, we only saw 10% ITC product formation, similar to plasma levels in clinical studies of cooked brassica. More recently an *ex vivo* study found that an 8-h incubation of glucoraphanin with human feces produced no ITC, whereas substantial amounts of sulforaphane and erucin were formed by 24 h (Saha et al., 2012). It is possible that this reflects an altered microbiome, by induction of an enzyme or growth of a specific bacterium during the 8–24 h period.

A number of studies have attempted to characterize specific gut bacteria that may be responsible for myrosinase-like activity, with the goal of determining how to enhance this activity, be it through bacterial growth or enzyme induction. Recently, an *E. coli* strain isolated from human feces was shown to hydrolyze sinigrin to allyl ITC, with ∼25% yield (Luang-In et al., 2015). Yet alone this is still not sufficient to provide *robust* hydrolysis within the gut. Whereas bacteria can support ITC formation, the extent of bacterial hydrolysis of glucosinolates is far less than by the specific plant myrosinase. Therefore we cannot yet fully explain the epidemiological findings of health benefits in persons eating 3–5 servings of brassica a week.

## Microbial Generation of ITC From Glucosinolates Depends Upon Frequency of Exposure

In designing clinical studies, subjects are typically given a list of brassica foods to avoid during the run-in of the study, so that the majority of glucosinolate bioavailability studies in the literature are the result of a single bolus of glucosinolate. In contrast to clinical studies, epidemiological studies do not regulate brassica intake. Furthermore, one study that did not ask people to avoid any foods prior to providing a glucosinolate-rich tea devoid of myrosinase, found urinary levels of sulforaphane metabolites varied between < 1% and 40% of the glucosinolate dose (Fang et al., 2013). Intriguingly, this study was carried out in both urban USA and rural China, with essentially the same spread in urinary sulforaphane metabolite outputs. This appears to exclude genotype or the larger environment as causes of this spread, but possibly not exclude variation in dietary intake of brassica over the preceding days. One possible explanation for these data is that both populations had variable exposure to brassica and that frequent exposure causes a change in the abundance/activity of the microbiome, enhancing microbial metabolism of glucosinolates. With this hypothesis in mind, we set out to determine if intake of brassica over multiple days (chronic intake) could alter metabolism of glucosinolates by the microbiome.

We carried out a study where, instead of a washout period before evaluating glucoraphanin hydrolysis by gut bacteria, we fed rats a 10% broccoli diet for a week. ITC can be estimated individually by GC, HPLC-DAD-MS, or as a group of parent ITC and metabolites by the cyclocondensation reaction (Zhang et al., 1992). In the cyclocondensation reaction, 1,2-benzenedithiol, binds the carbon of the ITC group whether it is free or part of a metabolite, to form stable 1,3-benzodithiole-2-thione, the endpoint of the assay. Although this allows estimation of total ITC, it does not identify whether the ITC is sulforaphane, its reduced congener erucin, metabolites of these, or even an unrelated thiocarbamate product from the microbiota. We specifically chose to use the cyclocondensation reaction to get a measure of total ITC formed, since both sulforaphane and the reduced product erucin might be formed, particularly given the anaerobic environment in which the microbiota are found.

**FIGURE 1:**
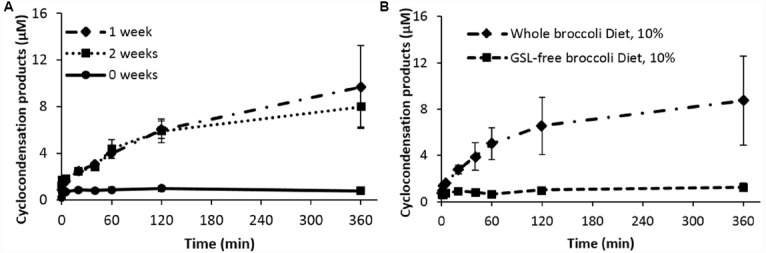
**Cyclocondensation products generated by ex vivo gut luminal microbiota hydrolysis of Glucoraphanin. (A)** Microbiota hydrolysis products from rats fed with 10% broccoli powder diet up to two weeks, in comparison to control diet. **(B)** Hydrolysis of glucoraphanin after incubation with luminal microbiota of rats that received 10% broccoli powder diet or 10% glucosinolate (GSL)-free broccoli powder diet. Results are the mean ± SD of three independent experiments. Each measurement is significantly different from control.

We fed rats (adult, male Fisher 344) an AIN93G-based diet containing 10% freeze-dried broccoli for 0, 1, or 2 weeks, before removing the caecal microbiota anaerobically to incubate them in broth in an anaerobic chamber. Separate incubates were removed from the chamber at 5 min to 6 h, treated with benzene dithiol and appearance of 1,3-benzodithiole-2-thione estimated by HPLC (Figure 1A). Because broccoli is rich in fiber, which can act as a prebiotic altering the microbiome, we repeated the study heating the broccoli powder in water at 80 °C overnight (glucosinolate-free broccoli, Figure 1B). Results clearly show that 1,3-benzodithiole-2-thione was formed in a time-dependent manner and that the formation was far greater after 1 week feeding glucosinolates. We detected no compound at time 0, showing there was no contamination from diet. A further week’s feeding made no significant difference from 1 week’s feeding. Furthermore, feeding a broccoli diet free of glucosinolates for a week had no impact, showing that the glucosinolates were the effective component. However, because we had used benzene dithiol to estimate product, we were unable to identify specific product(s).

## The Chemical Form that Permits Safe Transport of Isothiocyanates From Bacteria to Blood

**FIGURE 2:**
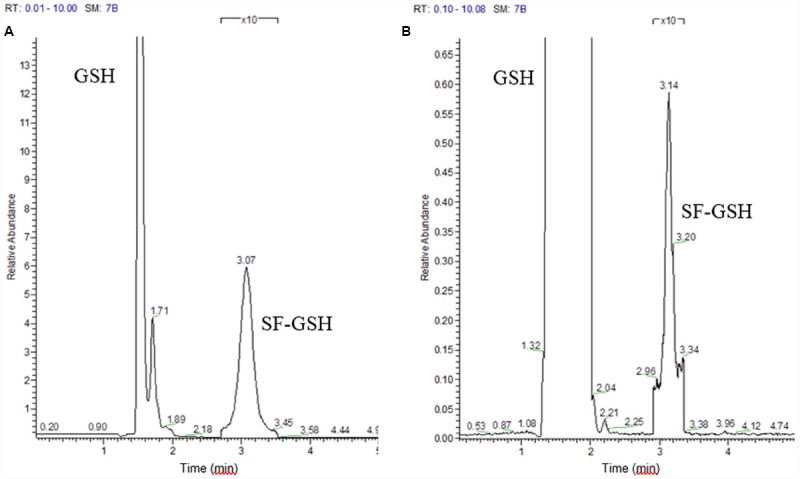
**Selected ion monitoring of (A) standard glutathione (GSH) and the sulforaphane-glutathione complex (SF-GSH); (B) Glutathione reaction with microbiota treated with glucoraphanin**.

Although the cyclocondensation method had provided an indication of increased ITC formation, analysis of the bacterial supernatant by GC or HPLC-MS revealed no ITC peak. Reviewing the binding properties of ITC, we considered that as a strong thiol, benzene dithiol might have extracted thiol-bound ITC. Therefore, we used an alternative thiol that would allow characterization of the product—and incubated with a great excess (10 mM) of GSH. Analyzing by LC-MS, we saw peaks for standard GSH, sulforaphane and a mix of the two that gave a unique GSH-sulforaphane peak (Figure 2A). Upon incubating the microbiota with GSH, we identified the GSH-sulforaphane peak (Figure 2B) and a second peak identified as GSH-erucin (data not shown). Knowing that ITC bind reversibly to protein-bound thiols, we hypothesize that by adding excess GSH, ITC were preferentially released from proteins where they had been cysteine-bound, binding to GSH for efflux from the cell. In some bacteria, as in mammalian cells, once GSH is bound to a xenobiotic, it is secreted and thus could become available for measurement, but only in a dynamic system that does not allow re-entry. In other bacteria, alternative small molecular weight thiols detoxify xenobiotic electrophiles such as bacillithiol would take the place of GSH (Fang et al., 2013; Loi et al., 2015).

## Conclusions and Future Directions

Since very little 1,3-benzodithiole-2-thione was formed during incubation of microbiota from rats that had not received the broccoli diet, we conclude that gut bacteria hydrolyze glucosinolates in the absence of plant myrosinase, but that they do so poorly unless pre-fed glucosinolate-containing foods, causing a change in the abundance and/or activity of microbiota with myrosinase-like activity.

For those bacteria in the microbiome that hydrolyze glucosinolates, once a strong electrophile such as the ITC sulforaphane has been formed, it will bind to intracellular components, only available for efflux when released by strong thiols such as benzene dithiol or GSH. Release by strong thiols would not be possible if the ITC had bound non-cysteine nucleophiles in the bacterium. Therefore, any ITC bound to non-thiol nucleophiles cannot be visualized by this method.

It remains to be determined (i) how gut microbial glucosinolate-hydrolyzing bacteria protect themselves from ITC, (ii) whether some of those bacteria that form ITC are killed by the ITC they produce, (iii) if some ITC that are released into the environment of the microbiome as thiol conjugates release free ITC that can kill other bacteria that have no protective system, or (iv) if the majority of the released ITC thiol conjugates are absorbed across the gut wall.

## Author Contributions

DA, EJ, and MM planned the study; DA and EJ carried out surgery; MVT and JLH carried out bacterial metabolisms; DA, ED, and JS measured product by cyclocondensation, HPLC and GC and LC-MS, respectively; PC, JMH, MM, and EJ interpreted data; AD and EJ wrote the article and all authors edited the article.

### Conflict of Interest Statement

The authors declare that the research was conducted in the absence of any commercial or financial relationships that could be construed as a potential conflict of interest.
